# ApoE Mimetic Peptide COG1410 Kills *Mycobacterium smegmatis* via Directly Interfering ClpC’s ATPase Activity

**DOI:** 10.3390/antibiotics13030278

**Published:** 2024-03-19

**Authors:** Chun Wang, Yun-Yao Ren, Li-Mei Han, Peng-Cheng Yi, Wei-Xiao Wang, Cai-Yun Zhang, Xiu-Zhen Chen, Ming-Zhe Chi, Apeng Wang, Wei Chen, Chun-Mei Hu

**Affiliations:** 1 Department of Tuberculosis, The Second Hospital of Nanjing, Affiliated to Nanjing University of Chinese Medicine, Nanjing 210003, China; 20211770@njucm.edu.cn (C.W.); 20211799@njucm.edu.cn (L.-M.H.); 20221893@njucm.edu.cn (P.-C.Y.); 2 Clinical Research Center, The Second Hospital of Nanjing, Affiliated to Nanjing University of Chinese Medicine, Nanjing 210003, China; 20221863@njucm.edu.cn (Y.-Y.R.); wangweixiao2021@njucm.edu.cn (W.-X.W.); zcaiyun8@163.com (C.-Y.Z.); xiuzhenchen2024@163.com (X.-Z.C.); 3 State Key Laboratory of Genetic Engineering, School of Life Science, Fudan University, Shanghai 200433, China; 22110700014@m.fudan.edu.cn; 4 Institute of Medicinal Biotechnology, Chinese Academy of Medical Sciences and Peking Union Medical College, Beijing 100050, China; wangapeng@imb.pumc.edu.cn

**Keywords:** antimicrobial peptide, ApoE, mimetic peptide, COG1410, resistance mechanism, ClpC, Clp protease, *Mycobacterium smegmatis*

## Abstract

Antimicrobial peptides (AMPs) hold promise as alternatives to combat bacterial infections, addressing the urgent global threat of antibiotic resistance. COG1410, a synthetic peptide derived from apolipoprotein E, has exhibited potent antimicrobial properties against various bacterial strains, including *Mycobacterium smegmatis*. However, our study reveals a previously unknown resistance mechanism developed by *M. smegmatis* against COG1410 involving ClpC. Upon subjecting *M. smegmatis* to serial passages in the presence of sub-MIC COG1410, resistance emerged. The comparative genomic analysis identified a point mutation in ClpC (S437P), situated within its middle domain, which led to high resistance to COG1410 without compromising bacterial fitness. Complementation of ClpC in mutant restored bacterial sensitivity. In-depth analyses, including transcriptomic profiling and in vitro assays, uncovered that COG1410 interferes with ClpC at both transcriptional and functional levels. COG1410 not only stimulated the ATPase activity of ClpC but also enhanced the proteolytic activity of Clp protease. SPR analysis confirmed that COG1410 directly binds with ClpC. Surprisingly, the identified S437P mutation did not impact their binding affinity. This study sheds light on a unique resistance mechanism against AMPs in mycobacteria, highlighting the pivotal role of ClpC in this process. Unraveling the interplay between COG1410 and ClpC enriches our understanding of AMP-bacterial interactions, offering potential insights for developing innovative strategies to combat antibiotic resistance.

## 1. Introduction

Drug-resistant tuberculosis poses a grave threat to global public health, exacerbating the burden of antimicrobial resistance and depleting substantial healthcare budgets and resources in many developing nations [1]. Regrettably, over the past decade, there has been no approval of novel drugs to combat multidrug-resistant TB (MDR-TB) or extensively drug-resistant TB (XDR-TB) except for Bedaquiline [2]. With *Mycobacterium tuberculosis* (MTB) rapidly developing antibiotic resistance, it becomes imperative to devise new anti-TB strategies to address the antimicrobial resistance crisis (AMR).

Antimicrobial peptides (AMPs) are short, predominantly cationic molecules found widely in nature [3]. They exert their potent antimicrobial effects by disrupting bacterial cell membranes or interacting with intracellular components, making them promising alternatives to traditional antibiotics [4]. AMPs have emerged as promising candidates for therapeutics to mitigate the escalating antimicrobial crisis [5], particularly in combating drug-resistant tuberculosis [6,7]. What sets AMPs apart are their unique mechanisms of action, which create a higher resistance barrier, reducing the likelihood of resistance development compared to conventional antibiotics [8,9]. Occasionally, AMPs also face challenges from bacteria. Assoni et al. summarized various resistance mechanisms to AMPs in Gram-positive bacteria, including alterations in cell envelope charge, extrusion by efflux pumps, and inhibition via binding to released capsular polysaccharides [10]. Remarkably, one of the most direct ways bacteria use to inactivate AMPs is by producing peptidases or proteases to degrade AMPs [11]. For instance, aureolysin, a protease produced by *Staphylococcus aureus*, can degrade human antimicrobial peptide LL-37 [12], and SerE, a serine protease in *Enterococcus faecalis*, can degrade GL13K, an AMP found in human saliva [13]. Therefore, proteases play a crucial role in protecting bacteria from AMPs’ attacks.

The caseinolytic protease (Clp) is a widely distributed multimeric degradation machinery found in bacteria, playing a crucial role in maintaining protein homeostasis and quality control [14]. Clp protease consists of the ATP-consuming hexameric Clp-ATPase (ClpC) and the barrel-shaped proteolytic core (ClpP) [15]. ClpC exhibits unfoldase activity and is responsible for substrate recognition, unfolding, and translocating the substrate to the degradation chamber of ClpP [16]. In mycobacteria, two unfoldases, ClpC and ClpX, associate with the proteolytic core ClpP, forming the active ClpCP or ClpXP protease, respectively [17,18,19]. In the context of the Clp system, the ATPases associated with diverse cellular activities (AAA+) play a key role in identifying and unfolding substrates. These substrates are then directed into the proteolytic chamber formed by barrel-shaped ClpP tetradecamers [20]. Further, these AAA+ ATPases are implicated in bacterial virulence, stress response, and genetic competence in cell physiology and differentiation [21,22,23,24,25,26]. The versatility of ClpC, coupled with its prevalence in Gram-positive bacteria, underscores its potential as a promising antibacterial drug target. Given its essential role in cell growth, ClpC has emerged as a promising target for anti-mycobacterial therapy.

Apolipoprotein E (ApoE), primarily associated with lipid metabolism within the central nervous system, harbors a range of cryptic bioactive peptides [27,28]. Some mimetic peptides derived from ApoE have demonstrated potent antimicrobial and immunomodulatory properties [29,30,31,32]. One such synthetic peptide is COG1410, comprising 12 amino acids, specifically residues 138–149 of the ApoE N-terminal domain with aminoisobutyric acid (Aib) substitutions at positions 140 and 145 [33]. In our previous study, we found that COG1410 effectively inhibited the growth of pandrug-resistant *Acinetobacter baumannii* [34] and *M. smegmatis* [27]. After 55 passages in the presence of sub-MIC COG1410, the MIC of COG1410 increased only 4-fold, compared to a 64-fold increase in polymyxin B [34]. However, in this study, using serial passages and genomic SNP analysis, we discovered that *M. smegmatis* developed significant resistance to COG1410, a resistance mechanism involving ClpC. Intriguingly, ClpC did not participate in AMP degradation via Clp protease but as an intracellular target of COG1410, which stimulated the ATPase activity of ClpC. Our study for the first time unveiled the mechanism of action for bactericidal activity of COG1410 and shed light on further refinement and enhancement of this anti-mycobacterial AMP.

## 2. Results

### 2.1. Induced Resistance of AMP Is Developed via Serial Passage in M. smegmatis

**Figure 1:**
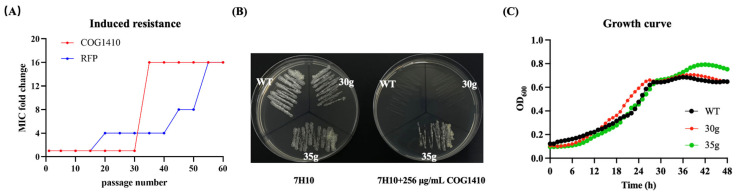
Resistance development of *M. smegmatis* in the presence of sub-MIC COG1410. (**A**) Bacterial resistance was induced via serial passage in the presence of sub-MIC antimicrobial agents. Rifampicin (RFP) was used as a positive control. (**B**) 35g strain exhibited high resistance against COG1410. (**C**) Resistance development did not sacrifice bacterial fitness. The growth curves of the wild-type strain and two mutants were determined in a plate reader.

After serial passage in the presence of sub-MIC COG1410 or rifampicin (RFP), *M. smegmatis* demonstrated significant resistance to both antimicrobial agents. Notably, resistance to RFP increased gradually, while resistance to the AMP emerged between the 30th and 35th generations, maintaining this level until the 55th generation without a further increase (Figure 1A). The MIC of COG1410 against *M. smegmatis* changed from 16 μg/mL to 256 μg/mL. To explore the underlying resistance mechanism, we isolated single colonies, respectively from the cultures of the 30th generation (30g) and 35th generation (35g) and determined their susceptibility to COG1410. MIC and MBC assays confirmed that 30g still remained sensitive to COG1410, while 35g developed resistance, with an MIC of 256 μg/mL and an MBC exceeding 256 μg/mL (Table 1 and Figure 1B). The growth of 30g and 35g was similar to that of the wild-type strain (Figure 1C), indicating that acquiring resistance did not compromise bacterial fitness.

### 2.2. Point Mutation of ClpC Is Involved in High Resistance of COG1410

Comparative genome analysis revealed that the 30g strain had 73 insertions or deletions and 16 single nucleotide polymorphisms (SNPs) compared to the wild-type strain. Similarly, the 35g strain had 72 insertions or deletions and 15 SNPs compared to the wild-type strain (Supplementary Materials, Tables S3 and S4). In comparison with 30g, 35g had 4 differences. Interestingly, 35g only exhibited an additional point mutation in *clpC* (MSMEG_RS29385), where a T was replaced with a C, resulting in the substitution of serine at position 437 with proline.

**Figure 2:**
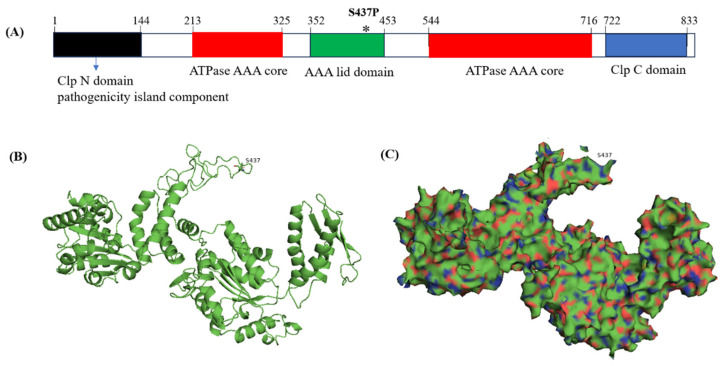
Schematic representation of AAA+ unfodase ClpC functional domains in *M. smegmatis*. (**A**) InterPro analysis shows that ClpC is composed of four domains. (**B**,**C**) Swiss model analysis reveals the protein structure of ClpC. The 437th serine is located at the coil-coil middle domain. * represents the location of the mutation site.

In *M. smegmatis*, ClpC consists of 848 amino acids, classified into five domains: Clp *N* domain (2–144), ATPase domain (213–325 and 544–716), Clp lid domain (352–453), and Clp C domain (722–813) (Figure 2A). The 437th serine is located within the middle lid domain of ClpC (Figure 2B). Complementation of ClpC, but not ClpC (S437P), into 35g restored bacterial sensitivity to COG1410 (Table 1). Therefore, we inferred that the point mutation of ClpC conferred resistance to COG1410 in *M. smegmatis*.

### 2.3. Knockdown of clpC Leads to COG1410 Resistance

**Figure 3:**
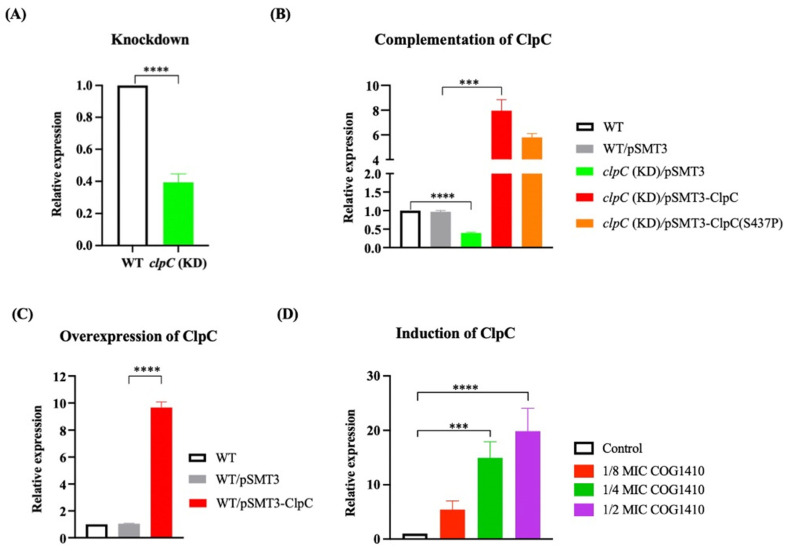
qRT-PCR analysis of expression level of ClpC. (**A**) *clpC* was suppressed using the CRISPRi method. (**B**) Complementation of ClpC in the *clpC* knockdown strain. (**C**) Overexpression of ClpC in the wild-type strain. (**D**) *clpC* was suppressed in the presence of COG1410. One-way ANOVA with multiple comparisons was used to analyze the differences between different groups and the control group. ***, *p* < 0.001; ****, *p* < 0.0001.

To elucidate whether ClpC is directly implicated in AMP resistance, we measured the MIC of COG1410 against *clpC* knockdown strains (kindly provided by Xue-Lian Zhang [35]). qPCR analysis indicated approximately a 50% suppression of *clpC* at the mRNA level (Figure 3A). Intriguingly, *clpC* (KD) strain displayed resistance to COG1410, with both MIC and MBC exceeding 256 μg/mL. Complementation of ClpC into the *clpC* (KD) strain elevated the expression level of ClpC (Figure 3B) and restored the MIC of COG1410 to 16 μg/mL (Table 1). Notably, the introduction of ClpC (S437P) did not reinstate bacterial sensitivity, suggesting that the point mutation of S437P in ClpC interfered with its normal function. Conversely, overexpression of ClpC in the wild-type strain did not confer resistance to COG1410 (Figure 3C and Table 1). These results indicate that ClpC, the unfoldase, is associated with COG1410 resistance in *M. smegmatis*.

### 2.4. ClpC Is Involved in Resistance of Other AMPs, but Not Conventional Antibiotics

To assess the specificity of ClpC’s interaction with COG1410, we evaluated the activity of two anti-TB AMPs, antiTB_1026 and antiTB_1080, sourced from the Anti-TB peptide database (AntiTbPdb), along with two ApoE mimetic AMPs, ApoE23 and COG133. All these synthetic short-cationic AMPs are recognized for their broad-spectrum antimicrobial activities against various bacterial pathogens [29,36]. Despite sharing similar sequences, COG133, one of the ApoE mimetic AMPs, did not demonstrate anti-mycobacterial activity, while ApoE23 exhibited bactericidal effects against *M. smegmatis* with a MIC of 128 μg/mL. Notably, both the 35g strain and the *clpC* (KD) strain displayed resistance to the two anti-TB AMPs despite their distinct sequences from COG1410 (Table 2). These results imply that ClpC may function as a shared intracellular target for cationic AMPs.

To address whether ClpC mutation affects the efficacy of conventional antibiotics, we selected several clinically relevant antibiotics known for their bactericidal activity against *M. smegmatis*, including azithromycin, amikacin, linezolid, ciprofloxacin, and cefoxitin. We determined their MIC and MBC against the wild-type strain, 35g strain, and *clpC* (KD) strain. Surprisingly, neither the point mutation nor knockdown of *clpC* had any discernible impact on the antimicrobial activity of these antibiotics (Table 3). These findings suggest that ClpC might exclusively play a role in the resistance to AMP and not affect the efficacy of conventional antibiotics.

### 2.5. ClpC Expression Is Induced by COG1410 in M. smegmatis

To investigate the impact of COG1410 on the expression of ClpC, we monitored the mRNA levels of *clpC* in the presence of different concentrations of COG1410. We observed a dose-dependent induction of *clpC* transcription upon the addition of COG1410. At 1/8 MIC (2 μg/mL) of COG1410, the mRNA level of *clpC* increased approximately fivefold compared to the control. Furthermore, at 1/2 MIC (8 μg/mL) of COG1410, bacterial growth was severely inhibited, and the mRNA level of *clpC* was enhanced up to 19-fold (Figure 3D). These findings suggest that *M. smegmatis* cells regulate the transcription of *clpC* in response to the threat posed by COG1410.

### 2.6. Transcriptome Analysis of M. smegmatis Response to COG1410

To comprehensively investigate the response of *M. smegmatis* to sub-MIC COG1410, we conducted a transcriptomic analysis using RNA-seq. Upon exposure to 1/8 MIC COG1410, 90 genes exhibited significant downregulation, while 36 genes displayed significant upregulation (Table S5). These differentially expressed genes were categorized into four types: metabolism, cellular process and signaling, information storage and processing, and poorly characterized. Notably, COG analysis revealed that the addition of COG1410 imposed a significant impact on genes associated with the transport and metabolism of amino acids, carbohydrates, and lipids (Table S5). Consistent with this, KEGG analysis highlighted the top three pathways affected by COG1410 energy metabolism, carbohydrate metabolism, and amino acid metabolism (Figure S1). Collectively, these findings strongly indicate that COG1410 causes the normal metabolic processes of *M. smegmatis* to be chaotic.

### 2.7. COG1410 Enters the Cytoplasm and Colocalizes with ClpC

**Figure 4:**
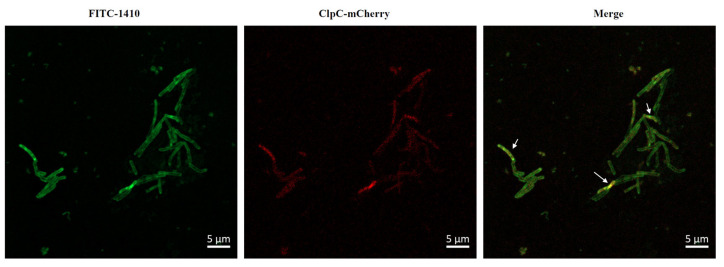
Colocalization of FITC-1410 and ClpC. FITC-1410 was incubated with *M. smegmatis* containing ClpC-mCherry expression plasmid and observed by Confocal microscopy. The white arrow indicates the overlap between COG1410 and ClpC. Scale bar, 5 μm.

To ascertain the relationship between COG1410 and ClpC, we decided to look into their localization. As expected, ClpC-mCherry displayed a diffuse distribution in the cytoplasm. However, even after incubation of 3 h, most FITC-1410 remained around the cell membrane, with only a small fraction penetrating the cytoplasm and colocalizing with ClpC (Figure 4). These data suggested that COG1410 is able to enter the cytoplasm of *M. smegmatis* and might interact with ClpC.

### 2.8. COG1410 Stimulates Its ATPase Activity

**Figure 5:**
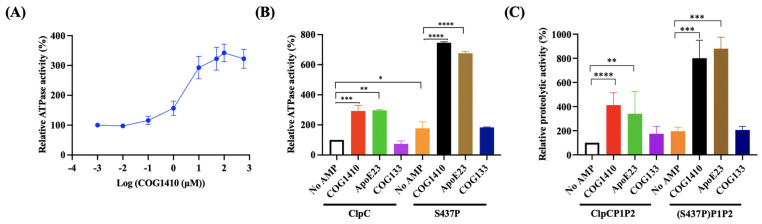
COG1410 interferes with ClpC’s activity. (**A**) COG1410 stimulates the ATPase activity of ClpC in a dose-dependent manner. (**B**) Addition of COG1410 enhances the ATPase activity of ClpC and exhibits a synergistic effect with S437P point mutation. (**C**) Addition of COG1410 enhances the proteolytic activity of the Clp protein and exhibits a synergistic effect with S437P point mutation. One-way ANOVA with multiple comparisons was used to analyze the differences between different groups and the no AMP control group. *, *p* < 0.05; **, *p* < 0.001; ***, *p* < 0.001; ****, *p* < 0.0001.

To investigate whether COG1410 directly influences the function of ClpC, we conducted in vitro assays to assess the ATPase activity of ClpC with or without COG1410. Interestingly, COG1410 significantly stimulated the ATPase activity of ClpC in a dose-dependent manner (Figure 5A). At 10 μM COG1410, the ATPase activity of ClpC increased approximately threefold compared to ClpC alone, a level comparable to that induced by another mycobacterium-killing AMP, ApoE23. In contrast, the addition of COG133 had no effect on ATPase activity, consistent with the ineffectiveness of COG133 against *M. smegmatis* (Figure 5B). These results suggest that COG1410 might exert its bactericidal effect on *M. smegmatis* by stimulating the ATPase activity of ClpC.

On the other hand, the ClpC (S437P) variant exhibited approximately a 1.6-fold increase in activity compared to its wild-type counterpart. Intriguingly, in the presence of 10 μM COG1410, the activity of ClpC (S437P) increased approximately 7.4-fold compared to ClpC alone. Similarly, the addition of ApoE23 also enhanced the ATPase activity of the ClpC variant (Figure 5B). These findings suggest that the S437P point mutation not only stimulates the ATPase activity of ClpC but also synergistically enhances its activity in the presence of COG1410.

### 2.9. COG1410 Stimulates the Proteolytic Activity of Clp Protease in M. smegmatis

To assess the impact of COG1410 on Clp proteolytic activity, we conducted in vitro proteolytic assays using FITC-casein. In the ClpCP1P2 system, the addition of 10 μM COG1410 led to a fourfold increase in proteolytic activity. Notably, the S437P variant of ClpC exhibited higher activity compared to the wild-type ClpC. Furthermore, consistent with the ATPase activity results, the addition of COG1410 in the S437PP1P2 system significantly enhanced protein degradation efficiency, reaching approximately sevenfold compared to the control with ClpC alone. ApoE23 exhibited a similar pattern to COG1410, showing a substantial increase in proteolytic activity. In contrast, COG133 had no influence on proteolytic activity (Figure 5C). In summary, COG1410 stimulated the proteolytic activity of Clp protease in *M. smegmatis*.

### 2.10. COG1410 Directly Binds with ClpC

**Figure 6:**
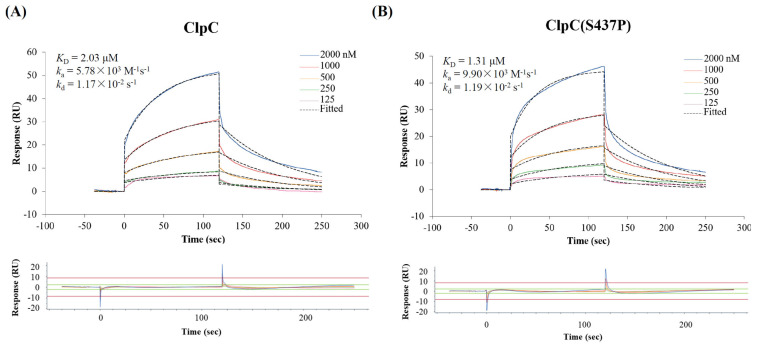
Confirmation of COG1410 binding with ClpC (**A**) and ClpC (S437P) (**B**) by SPR.

**Figure 7:**
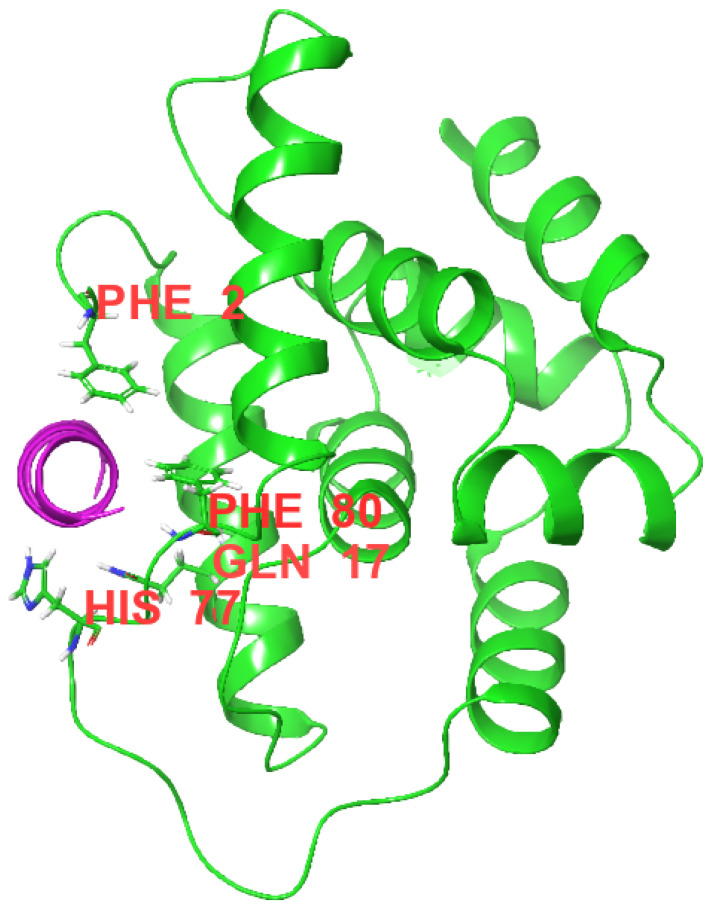
Image of a Docking analysis. COG1410 (purple) binds with *M. smegmatis* ClpC at the N-domain (green). The corresponding residues involved in the interaction were labeled.

Surface plasmon resonance (SPR) experiments showed that COG1410 binds to ClpC with a K_D_ of 2.03 μM (Figure 6). The association constant (k_a_) and dissociation constant (k_d_) were 5.78 × 10^3^ M^−1^s^−1^ and 1.17 × 10^−2^ s^−1^, respectively. To our surprise, the S437P mutation exhibited comparable affinity with COG1410, with a K_D_ of 1.31 μM, k_a_ of 9.9 × 10^3^ M^−1^s^−1^, and k_d_ of 1.19 × 10^−2^ s^−1^. Docking analysis showed that COG1410 bonded with ClpC at its N terminus and interacted with a few residues, including Phe 2, Gln 17, His 77, Phe 80, and Lys 85 (Figure 7). Therefore, these data suggest that COG1410 directly binds to ClpC, and the S437P mutation does not affect binding.

## 3. Discussion

AMPs present a promising solution to the pressing issue of antimicrobial resistance in the face of bacterial pathogens. Understanding bacterial resistance mechanisms against AMPs proves beneficial in revealing their mechanisms of action, as well as further refinement and enhancement. COG1410 has garnered significant acclaim for its neuroprotective and anti-inflammatory effects in vitro and clinically relevant models of brain injury [33,37]. Notably, recent reports highlight COG1410’s capacity to mitigate blood–brain barrier injury in a rat model of ischemic stroke [38]. In cellular contexts, COG1410 is established to bind to the SET protein, a potent physiological inhibitor of protein phosphatase 2A (PP2A). This interaction enhances endogenous PP2A phosphatase activity, leading to a reduction in levels of phosphorylated kinases, signaling, and inflammatory responses [39]. In this study, for the first time, we identified ClpC as the intracellular target of COG1410 in bacteria. Previously, we observed COG1410’s broad-spectrum antimicrobial activity, primarily centered on disrupting the integrity of bacterial cell membranes. Compared with untreated cells, the release of cell contents increased transparency in the treated cells in *M. smegmatis* [27]. Meanwhile, we noticed that the major part of COG1410 stagnated around the cell membrane. Therefore, we hypothesize that COG1410 may employ a dual mechanism, targeting both the bacterial cell membrane and ClpC, to achieve its bactericidal activity.

Given the Clp system’s imperative role, bacteria usually regulate their proteolytic activity precisely [15]. Deviations, whether toward suppression or enhancement of this activity, pose threats to bacterial survival. For example, the natural cyclic peptide cyclomarin A targets ClpC1 in *M. tuberculosis*, interfering with its ATPase function to induce excessive proteolysis [40]. In contrast, lassomycin, another anti-mycobacterial AMP, targets ClpC1 to stimulate its ATPase activity, uncoupling it from ClpP proteolysis [41]. Recently, a novel group of AMPs called rufomycins was reported to bind with ClpC1, decreasing the proteolytic capabilities of the Clp protease without significantly affecting the ATPase activity of ClpC1 [42]. In our study, COG1410 represents a novel mechanism of action, which binds with ClpC, stimulates its ATPase activity, and enhances ClpCP1P2-catalyzed protein breakdown. Consistently, transcriptome analysis confirmed that the addition of COG1410 interferes with the normal metabolic processes of *M. smegmatis*.

On the other hand, it is noteworthy that COG1410 stimulated the transcription of *clpC*. To the best of our knowledge, this is the first report on the regulation of *clpC* at the transcriptional level. Previous studies by Taylor et al. indicated that ClpC levels remained relatively constant over an 8 h timeframe in *M. smegmatis*. They also reported that the ClpC analog, ClpC2, is inducible when exposed to AMPs [43]. Thus, we hypothesize that the expression level of *clpC* is subject to regulation besides its activity. COG1410 not only interferes with ClpC’s activity but also affects its expression level. The regulatory mechanisms through which ClpC modulates its own expression require further investigation.

The N-terminal domain (NTD) of ClpC1 in *M. tuberculosis* is known to interact with adaptor/regulatory proteins responsible for modulating Clp protease activity [44,45]. Mutations in the NTD of ClpC1, resulting in resistance to certain AMPs, have been previously documented [41,42]. Our study is the first to report a mutation in the middle domain of ClpC that confers resistance to anti-mycobacterial AMPs. The 437th serine, located in the middle domain, is implicated in this resistance mechanism. Cryo-EM analysis has revealed that four ClpC hexamers interact via their coiled-coil M domains, forming a network of helix pairs that hold the particle together [46]. These findings suggest that the middle domain plays a crucial role in maintaining ClpC’s normal function. The intricate regulation and functional dynamics of the Clp protease system, particularly the central role played by ClpC and its interaction with AMPs, offer valuable insights that not only contribute to our understanding of bacterial physiology but also hold promise for the targeted development of antibacterial drugs.

In our study, there are several intriguing observations and paradoxes that need further investigation and clarification. At first, we observed that the spontaneous mutant, 35g strain, was resistant to COG1410 and exhibited a growth curve similar to that of the wild-type strain. This suggests that the S437P mutation of ClpC does not adversely affect cell growth but rather demonstrates an antagonistic effect against COG1410’s bactericidal activity. Compared with ClpC alone, ClpC (S437P) exhibited an approximately 1.6-fold increase in activity. In the presence of COG1410, the ATPase activity of ClpC and ClpC (S437P) increased by about 3-fold and 7.4-fold, respectively. If the mechanism of action of COG1410 involves excessive proteolysis of Clp protease by stimulating ClpC’s ATPase activity, binding of COG1410 with ClpC (S437P) should lead to more death, given its higher ATPase activity. However, the actual situation is the opposite, with the strain containing ClpC (S437P) showing resistance to COG1410. On the other hand, the knockdown of *clpC* conferred resistance to COG1410, indicating that ClpC is necessary for COG1410’s bactericidal effect. SPR assays confirmed that COG1410 directly binds to ClpC. In other words, ClpC is the target of COG1410 in vivo. The point of contention arises from the fact that when the amount of ClpC in the cytoplasm is reduced, less COG1410 is needed to interfere with the remaining ClpC’s normal function, making the bacterium more sensitive to COG1410. Similarly, overexpression of ClpC would accumulate more ClpC, requiring more COG1410 to deal with the surplus ClpC. One example is ClpC2, which absorbs more compounds like a sponge to counteract the toxic effects of AMP [43]. However, the actual situation contradicts this expectation, as overexpression of ClpC did not affect the MIC of COG1410. Therefore, how S437P mutation leads to resistance warrants further investigation.

In conclusion, we identified the intracellular target of COG1410 in *M. smegmatis*, which directly binds with ClpC and stimulates its ATPase activity. Despite the similarities between ClpC1 in *M. tuberculosis* and ClpC in *M. smegmatis*, COG1410 did not exhibit bactericidal activity against *M. tuberculosis*. We hypothesize that this discrepancy may be attributed to the thicker cell walls and reduced porins in the *M. tuberculosis* membrane, hindering COG1410 entry. Uncovering the direct target of COG1410 represents a significant advancement that holds the potential to expedite the development of COG1410 derivatives. These derivatives could overcome the challenges posed by the robust cell walls in *M. tuberculosis*, paving the way for more effective antimicrobial strategies.

## 4. Methods and Materials

### 4.1. Bacterial Strains and Growth Conditions

Bacterial strains and plasmids used in this study are listed in Table S1. The *Mycobacterium smegmatis* strains were grown in Middlebrook 7H9 broth (BD Bioscience, San Diego, CA, USA) supplemented with 10% Middlebrook oleic acid dextrose and catalase enrichment (OADC, (BD Bioscience, San Diego, CA, USA)), 2% (*w*/*v*) glycerol (Aladdin, Shanghai, China), and 0.08% (*v*/*v*) Tween80 (Biosharp, Anhui, China) at 37 °C without shaking, or streaked on the Middlebrook 7H10 agar (BD Bioscience, San Diego, CA, USA) supplemented with 10% OADC. The *E. coli* strains were routinely cultured in LB medium or on LB agar at 37 °C. When necessary, 100 μg/mL hygromycin (ACMEC, Shanghai, China) or 50 μg/mL carbenicillin (Sango, Shanghai, China) was supplemented in the media.

### 4.2. Peptides

All the peptides were purchased from GL Biochem (Shanghai, China) Ltd., with purity over 95%. The trifluoroacetic acid (TFA) was replaced by acetate, and the remaining TFA was less than 1%. The amino acid sequences of peptides used in this study are listed in Table 2.

### 4.3. Plasmid Construction and Transformation

All oligo primers used in this study are listed in Table S2. To construct the expression plasmid for ClpC or ClpC (S437P) in *M. smegmtis*, the shuttle vector pSMT3 was linearized by restriction with *Bam*HI and *Hind*III. Subsequently, *clpC* and *clpC* (S437P) were amplified using the primers clpC-F and clpC-R from the genomic DNAs of the wild-type strain and the 35g strain, respectively. The resulting fragments were purified and then fused with the linearized pSMT3 using the In-Fusion^®^ HD Cloning Kit (Takara Bio, Beijing, China), generating pSMT3-clpC and pSMT3-clpC (S437P). To express ClpC, ClpC (S437P), ClpP1, and ClpP2, their open reading frames (ORFs) were amplified with respective primers from the genomic DNAs of the wild-type strain and the 35g strain. These fragments were then cloned into pET28a using the In-Fusion assay. All plasmids were confirmed via sequencing.

The transformation of *M. smegmatis* with pSMT3 and its derivatives was accomplished through electroporation, as previously described [47]. Confirmation of all transformants was conducted using colony PCR and sequencing.

### 4.4. Drug-Resistance Development Assay

The drug-resistance development assay was conducted in accordance with our previously established protocol [27]. In brief, spontaneous AMP-resistant mutants of *M. smegmatis* were generated by initially exposing the culture of the wild-type strain to sub-lethal MIC of COG1410. Subsequently, a small volume of the culture was extracted daily and introduced into a fresh medium containing COG1410. The concentrations of COG1410 were incrementally doubled every ten passages, and the cultures were harvested every five passages over a span of 60 passes. Rifampicin served as the positive control for drug resistance. The susceptibility of the collected bacteria to COG1410 and rifampicin was assessed using micro-dilution methods. The fold change in MIC was recorded and analyzed.

#### 4.4.1. Minimal Inhibitory Concentration (MIC) and Minimal Bactericidal Concentration (MBC)

The log-phase cultures of mycobacteria were prepared, diluted, and dispensed into 96-well plates, with each well containing a final McFarland (MCF) unit of 0.05 (approximately 10^5^ CFU/mL) and 100 μL per well, with four wells allocated for each sample. Various concentrations of antimicrobial agents were prepared via a 2-fold gradient dilution and added to the wells in equal volumes. Drug-free medium served as the growth control. Medium without bacteria served as a negative control. The plates were sealed with parafilm and incubated at 37 °C for 48 h. Optical density (OD) values were measured spectrophotometrically at 600 nm. Simultaneously, for MBC determination, 10 μL of culture was extracted from each well, dropped onto 7H10 agar, and incubated at 37 °C for 48 h. The MIC is defined as the minimum concentration of antimicrobial agents that inhibits 90% of bacterial growth, while the MBC is defined as the minimum concentration that completely inhibits bacterial growth. Both MIC and MBC assays were conducted in triplicate.

#### 4.4.2. Genomic SNP/InDel Analysis

To analyze the putative mutations responsible for AMP resistance, genome sequencing was conducted on the wild-type strain, 30 g and 35 g, followed by SNP/InDel analysis. Genomic DNAs were extracted from each strain, and sequencing libraries were prepared using the NEBNext^®^ Ultra™ DNA Library Prep Kit for Illumina (NEB, Ipswich, MA, USA) according to the manufacturer’s recommendations. The entire genomes of three *M. smegmatis* strains were sequenced using Illumina NovaSeq PE150 at Beijing Novogene Bioinformatics Technology Co., Ltd. SNP/InDel (Joint Calling) Detection GATK 4.0.5.1 was employed for the detection of individual SNPs, as well as insertions and deletions of small fragments (<50 bp). Additionally, the analysis of SNP/InDel variations in functional regions of the genome was performed. The raw data from the genome analysis were deposited in the SRA database with accession number PRJNA1030525.

#### 4.4.3. qRT-PCR

The exponential phase culture was harvested, and the total RNA was extracted using RNAprep Pure Cell/Bacteria Kit (TIANGEN (Beijing, China), Cat. No. DP430), which was converted to cDNA by reverse transcription using HiScript II Q RT SuperMix for qPCR (+gDNA wiper) (Vazyme (Nanjing, China), Cat. No. R223-01). qRT-PCR was performed to measure the expression level of *clpC* using ChamQ SYBR qPCR Master Mix (Vazyme, Cat. No. Q311-02) in the ABI 7500 Real-Time system. The qPCR conditions were as follows: denaturation steps at 95 °C for 30 s, followed by 40 cycles including denaturation at 95 °C for 10 s, annealing at 60 °C for 30 s, and extension at 60 °C for a duration of 60 s. *M. smegmatis* 16S rDNA gene served as the internal control for normalization. Three replications were set up for each sample. The relative expression levels of the target gene were determined using the 2^−ΔΔCt^ method. Three independent experiments were conducted. The results were presented as mean ± SD.

To determine the effect of COG1410 on the transcription level of *clpC*, different concentrations of COG1410 were added to the log-phase culture of *M. smegmatis* WT strain and incubated for 2 h, and then the cultures were harvested for qPCR.

#### 4.4.4. RNA-Seq Analysis

To determine the response of *M. smegmatis* to the presence of COG1410, 1/8 MIC COG1410 was added to the log-phase culture of *M. smegmatis* WT strain and incubated for 2 h. Three replicates were set up for each sample. The cultsseures were harvested using centrifugation, frozen in liquid nitrogen, and shipped with dry ice to Shanghai Majorbio Bio-pharm Technology Co. Ltd. (Shanghai, China). Total RNA was extracted from the tissue using the CTAB method, and genomic DNA was removed. Only a high-quality RNA sample was used to construct the sequencing library, which was sequenced using the Illumina Novaseq 6000 (Illumina Inc., San Diego, CA, USA). All of the analyses were performed using the free online platform of Majorbio Cloud Platform (https://www.majorbio.com, accessed on 9 March 2024). The differentially expressed genes were identified using the edgeR. Goatools (https://github.com/tanghaibao/GOatools, accessed on 9 March 2024) is used to identify statistically significantly enriched GO terms using Fisher’s exact test. KOBAS 2.0 (http://kobas.cbi.pku.edu.cn, accessed on 9 March 2024) is used to identify statistically significantly enriched pathways using Fisher’s exact test. The raw data have been deposited to the SRA database with the accession number PRJNA1056343.

#### 4.4.5. Determination of Co-Localization between COG1410 and ClpC

To investigate the entry of COG1410 into the cytoplasm and its colocalization with ClpC, 2 μg/mL (1/8 MIC) FITC-labeled COG1410 was applied to treat *M. smegmatis* containing pSMT3-clpC-mCherry. Cultures were sampled at various time points, and 5 μL of each culture was spotted on a clean slide coated with a thin layer of 1% agarose. Fluorescence was observed using a Confocal Microscope ZEISS LSM 900 (ZEISS, Oberkochen, Germany). For FITC, excitation and emission were set at 488 nm and 525 nm, respectively. For mCherry, the excitation and emission were set at 587 nm and 610 nm, respectively.

#### 4.4.6. Protein Purification for Functional Assay

The plasmids pET28a-ClpC, pET28a-ClpC (S437P), pET28a-ClpP1, and pET28a-ClpP2 were transformed into *E. coli* BL21 (DE3) (Novagen, Beijing, China) and cultivated in LB medium supplemented with 30 μg/mL kanamycin. Recombinant protein expression was induced by the addition of IPTG, and the proteins were subsequently purified using immobilized metal affinity chromatography (IMAC) using the Ni Sepharose™ 6 FF column in the ÄKTA avant chromatography system. The collected proteins were subjected to buffer exchange, including 20 mM Tris-HCl (pH 7.4) and 50 mM NaCl, using a desalting column. The purity of the proteins was monitored via SDS-PAGE, and the protein concentration was determined using the Bradford Protein Assay Kit (Beyotime (Haimen, China), Cat. No. P006).

#### 4.4.7. In Vitro ATPase Activity Assay

The ATPase activity of purified ClpC and ClpC (S437P) was assessed using the ATPase activity detection kit (Sangon (Shanghai, China), Cat. No. D799642-0100), following the manufacturer’s instructions. ATPase catalyzes the hydrolysis of ATP, producing ADP and inorganic phosphate as byproducts. In this kit, the generated inorganic phosphate was quantified to determine ATPase activity. Each reaction included 64 nM ClpC or ClpC (S437P). To evaluate the impact of COG1410 on ATPase activity, various concentrations of COG1410 were introduced into the reaction. ApoE23 and COG133 served as controls. The experiments were conducted in triplicate, and the results were presented as mean ± SD.

#### 4.4.8. In Vitro Protease Activity Assay

The proteolytic activity assay was conducted following a previously established protocol [42,48]. The reaction buffer consisted of 100 mM Tris, 200 mM KCl, and 8 mM MgCl_2_ (pH 7.5), with a total volume of 100 μL. In the reaction mixture, the final concentrations of ClpC and ClpC (S437P) were set at 1 μM, while ClpP1 and ClpP2 were maintained at 2 μM. Additionally, 100 μM ATP and 1 μM FITC-casein (Sigma-Aldrich (St. Louis, MO, USA), Cat. No. C0528) were added. To evaluate the impact of COG1410 on Clp proteolytic activity, various concentrations of COG1410 were introduced into each reaction. Six replicates were established for each treatment in the 96-well plate. ApoE23 and COG133 were served as controls. The reaction was incubated for 1 h at 37 °C, and green fluorescence was measured with excitation at 485 nm and emission at 535 nm. The experiments were conducted in triplicate, and the results were presented as mean ± SD.

#### 4.4.9. SPR Assay

Surface plasmon resonance (SPR) experiments were performed using a Biacore 8K (GE Healthcare, Chicago, IL, USA). 20 μg/mL recombinant protein ClpC and its variant ClpC (S437P) were immobilized onto a Series S Sensor Chip CM5 (GE Healthcare) with Amine Coupling Kit (GE Healthcare) at pH 4.0 (10 μL/min, both around 6500 RU). The binding of COG1410 was measured with a continuous flow of PBS, pH 7.4, 0.05% tween-20 at 25 °C (30 μL/min, association 120 s, dissociation 120 s, additional 30 s for regeneration). The 2-fold serial dilutions of COG1410 were flowed through with a concentration ranging from 2000 to 125 nM. All the binding data were double-referenced by blank cycle and reference flow cell subtraction. The resulting data fit a 1:1 Langmuir binding model using Biacore Insight Evaluation Software 4.0 (GE Healthcare).

#### 4.4.10. Docking

To further understand the interactions between 1410 and ClpC, an in silico procedure was performed. The residues 138–149 of ApoE (PDB: 1B68) were extracted using PyMOL 2 software and then opened in Schrodinger 2023-4 software. The residues 140 and 145 were replaced by aminoiso-butyric acid (Aib) along with the acylation of the N terminus and amidation of the C terminus. The structure of the *M. tuberculosis* ClpC1 N-domain was obtained using the Protein Data Bank (PDB: 3WDB). Schrodinger 2023-4 software was used to perform the peptide-protein docking runs.

#### 4.4.11. Statistical Analysis

Statistical analysis was performed using GraphPad Prism software 9.3.0. The statistical differences among different groups were assessed using One-way ANOVA with multiple comparisons.

## Figures and Tables

**Table 1:** Minimal inhibitory concentration (MIC) and minimal bactericidal concentration (MBC) of COG1410 against different *M. smegmatis* strains.

Strains	MIC (µg/mL)	MBC (µg/mL)
WT	16	32
30g	16	32
35g	256	>256
35g/pSMT3	256	>256
35g/pSMT3-ClpC	16	64
35g/pSMT3-ClpC (S437P)	256	>256
*clpC* (KD)	>256	>256
*clpC* (KD)/pSMT3	>256	>256
*clpC* (KD)/pSMT3-ClpC	8	16
*clpC* (KD)/pSMT3-ClpC (S437P)	>256	>256
WT/pSMT3	16	32
WT/pSMT3-ClpC	16	32

Note: WT, wild type; KD, knockdown.

**Table 2:** MIC of other AMPs against different *M. smegmatis* strains.

Peptides	WT	35g	*clpC* (KD)	Sequences	Sources
COG1410	16	256	256	AS(Aib)LRKL(Aib)KRLL	[31]
ApoE23	128	>128	>128	LRKLRKRLVRLASHLRKLRKRLL	[35]
COG133	>256	>256	>256	LRVRLASHLRKLRKRLL	[28]
antiTB_1026	32	256	>256	WKWLKKWIK	[34]
antiTB_1080	32	128	>256	IRMRIRVLL	[34]

Note: Aib, 2-Aminoisobutyric acid; KD, knockdown.

**Table 3:** MIC of conventional antibiotics against *M. smegmatis* mutants.

Antibiotics	WT	35g	*clpC* (KD)
azithromycin	2	2	2
amikacin	0.5	0.5	0.5
linezolid	1	1	1
ciprofloxacin	0.5	0.5	0.5
cefoxitin	16	16	16

Note: KD, knockdown.

## Data Availability

Data are contained within the article and Supplementary Materials.

## Supplementary Materials

The following supporting information can be downloaded at: https://www.mdpi.com/article/10.3390/antibiotics13030278/s1, Table S1. Strains and plasmids in this study; Table S2. Primers used in this study; Table S3. InDel differences between the wild-type strain and induced mutants; Table S4. SNP differences between the wild-type strain and induced mutants; Table S5. Transcriptome analysis of *M. smegmatis* treated with sub-MIC COG1410; Figure S1. KEGG analysis of DEGs affected by COG1410 in *M. smegmatis*. Reference [49] is mentioned in the Supplementary Materials.
