# Supplementary material for: ApoE Mimetic Peptide COG1410 Kills Mycobacterium smegmatis via Directly Interfering ClpC’s ATPase Activity
Source: Antibiotics (Basel). 2024 Mar 19;13(3):278. doi: 10.3390/antibiotics13030278 (PMC10967448; doi:10.3390/antibiotics13030278)
Supplement: Supplementary file 1 [file antibiotics-13-00278-s001.zip › Table S1. Strains and plasmids in this study..pdf]

Table S1. Strains and plasmids used in this study.

Strain or Plasmid	Genotypes or Relative Characteristics	References or Sources
**Strains**		
Mycobacterium smegmatis MC²-155	the wild-type strain (WT)	lab storage
30g	spontaneous mutant of *M. smegmatis*, COG1410 sensitive	this study
35g	spontaneous mutant of *M. smegmatis*, COG1410 resistant	this study
35g/pSMT3-clpC	expression of ClpC in 35g strain, hygromycin resistant	this study
35g/pSMT3-clpC (S437P)	expression of ClpC (S437P) in 35g strain, hygromycin resistant	this study
clpC (KD)	*clpC* knockdown strain in *M. smegmatis*	[1]
clpC (KD)/pSMT3	*clpC* knockdown strain containing empty vector, hygromycin resistant	this study
clpC (KD)/pSMT3-clpC	expressing ClpC in the *clpC* knockdown strain, hygromycin resistant	this study
clpC (KD)/pSMT3-clpC (S437P)	expressing ClpC (S437P) in the *clpC* knockdown strain, hygromycin resistant	this study
WT/pSMT3	the wild-type strain containing empty vector, hygromycin resistant	this study
WT/pSMT3-clpC	overexpression of ClpC in the wild-type strain, hygromycin resistant	this study
**Plasmids**		
pSMT3	shuttle vector for mycobacteria, hygromycin resistant	[2]
pSMT3-clpC	ClpC expression vector in mycobacteria, hygromycin resistant	this study
pSMT3-clpC (S437P)	ClpC (S437P) expression vector in mycobacteria, hygromycin resistant	this study
pSMT3-clpC-mCherry	expression vector of ClpC-mCherry fusion protein, hygromycin resistant	this study
pSMT3-P'clpC-mCherry	clpC promoter reporter plasmid, hygromycin resistant	this study
pET15b	protein expression vector, ampicillin resistant	lab storage
pET15b-clpC	His-tagged ClpC expression vector, ampicillin resistant	this study
pET15b-clpP1	His-tagged ClpP1 expression vector, ampicillin resistant	this study
pET15b-clpP2	His-tagged ClpP2 expression vector, ampicillin resistant	this study

1 Bai, J.-C.; Chi, M.-Z.; Hu, Y.-W.; Hao, M.; Hao, X.-L. Construction and Biological Characteristics of ClpC and ClpX Knock-down Strains in *Mycobacterium smegmatis*. *China Biotechnol.* **2021**, *41*, 13–22

2 Carroll, P.; Schreuder, L.J.; Muwanguzi-Karugaba, J.; Wiles, S.; Robertson, B.D.; Ripoll, J.; Parish, T. Sensitive detection of gene expression in mycobacteria under replicating and non-replicating conditions using optimized far-red reporters. *PLoS ONE* **2010**, *5*, e9823.
