# Supplementary figures and images for: ApoE Mimetic Peptide COG1410 Kills Mycobacterium smegmatis via Directly Interfering ClpC’s ATPase Activity
Source: Antibiotics (Basel). 2024 Mar 19;13(3):278. doi: 10.3390/antibiotics13030278 (PMC10967448; doi:10.3390/antibiotics13030278)

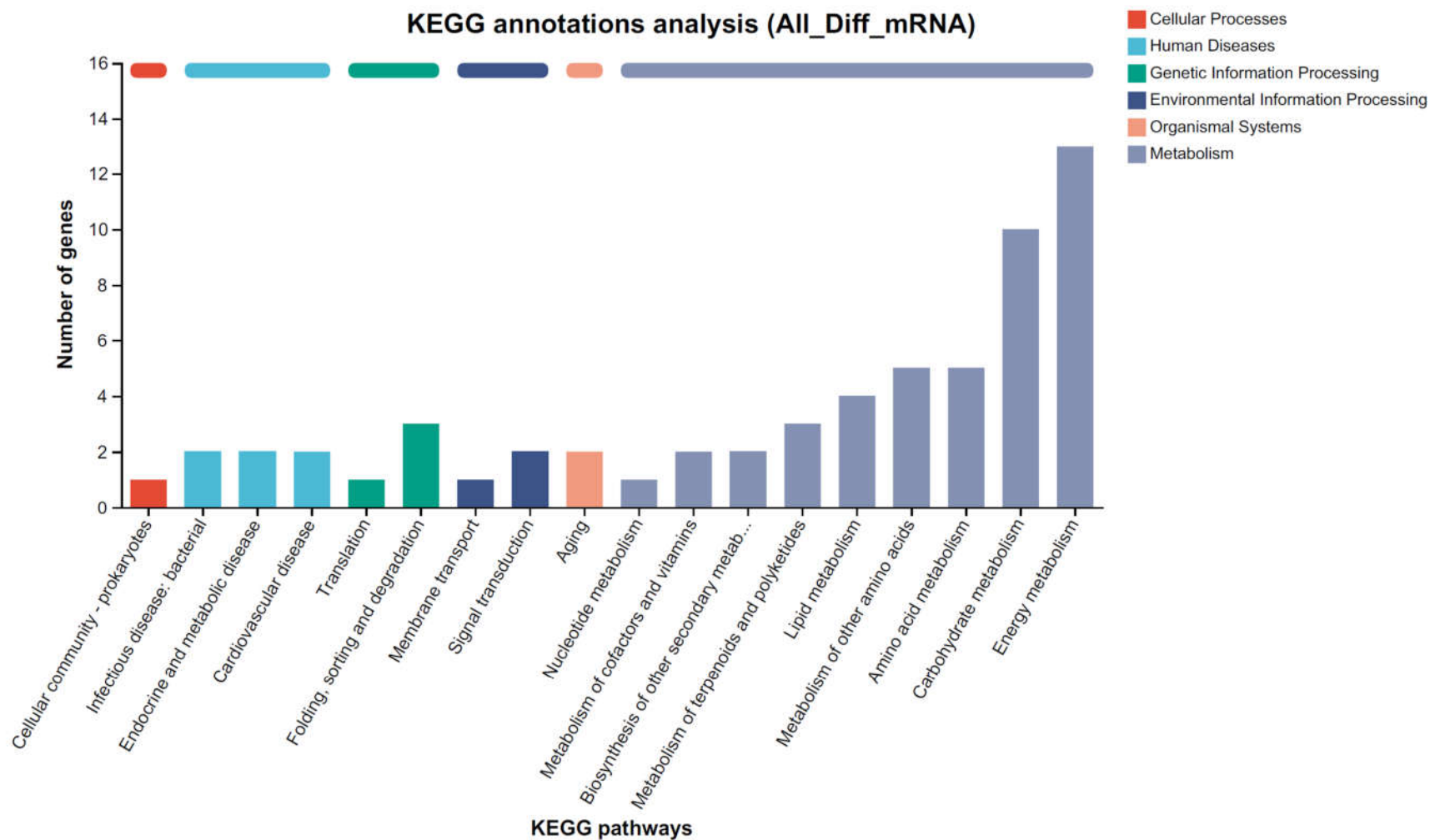

**Figure S1.** KEGG analysis of DEGs affected by COG1410 in *M. smegmatis*.

Supplement: Supplementary file 1 [file antibiotics-13-00278-s001.zip › Figure S1. KEGG analysis of DEGs affected by COG1410 in M. smegmatis..pdf]
